# Burst Strength of BIOLOX*^®^*delta Femoral Heads and Its Dependence on Low-Temperature Environmental Degradation

**DOI:** 10.3390/ma13020350

**Published:** 2020-01-12

**Authors:** Toshiyuki Tateiwa, Elia Marin, Alfredo Rondinella, Marco Ciniglio, Wenliang Zhu, Saverio Affatato, Giuseppe Pezzotti, Ryan M. Bock, Bryan J. McEntire, B. Sonny Bal, Kengo Yamamoto

**Affiliations:** 1 Department of Orthopaedic Surgery, Tokyo Medical University, 6-7-1, Nishishinjuku, Shinjuku-ku, Tokyo 160-0023, Japan; tateiwa@tokyo-med.ac.jp (T.T.); pezzotti@kit.ac.jp (G.P.); 2 Ceramic Physics Laboratory, Kyoto Institute of Technology, Sakyo-ku, Matsugasaki, Kyoto 606-8585, Japan; marcociniglio@libero.it (M.C.); wlzhu@kit.ac.jp (W.Z.); 3 Department of Dental Medicine, Graduate School of Medical Science, Kyoto Prefectural University of Medicine, Kamigyo-ku, Kyoto 602-8566, Japan; 4 DPIA, University of Udine, 33100 Udine, Italy; al.rondinella@gmail.com; 5 Laboratorio di Tecnologia Medica, IRCCS Istituto Ortopedico Rizzoli, 40136 Bologna, Italy; affatato@tecno.ior.it; 6 Department of Immunology, Graduate School of Medical Science, Kyoto Prefectural University of Medicine Kamigyo-ku, 465 Kajii-cho, Kawaramachi dori, Kyoto 602-0841, Japan; 7 The Center for Advanced Medical Engineering and Informatics, Osaka University, Yam-adaoka, Suita, Osaka 565-0871, Japan; 8 SINTX Corporation, Salt Lake City, UT 84119, USA; rbock@sintx.com (R.M.B.); BMcEntire@sintx.com (B.J.M.); SBal@sintx.com (B.S.B.)

**Keywords:** burst strength, BIOLOX^®^delta, femoral head, Raman microprobe spectroscopy

## Abstract

Zirconia-toughened alumina (ZTA) currently represents the bioceramic gold standard for load-bearing components in artificial hip joints. ZTA is long known for its high flexural strength and fracture toughness, both properties arising from a microscopic crack-tip shielding mechanism due to the stress-induced tetragonal-to-monoclinic (t→m) polymorphic transformation of zirconia. However, there have been concerns over the years regarding the long-term structural performance of ZTA since the t→m transformation also spontaneously occurs at the material’s surface under low-temperature environmental conditions with a concomitant degradation of mechanical properties. Spontaneous surface degradation has been extensively studied in vitro, but predictive algorithms have underestimated the extent of in vivo degradation observed in retrievals. The present research focused on burst-strength assessments of Ø28 mm ZTA femoral before and after long-term in vitro hydrothermal ageing according to ISO 7206-10. An average burst strength of 52 kN was measured for pristine femoral heads. This value was ~36% lower than results obtained under the same standard conditions by other authors. A further loss of burst strength (~13% in ultimate load) was observed after hydrothermal ageing, with increased surface monoclinic content ranging from ~6% to >50%. Nevertheless, the repetitively stressed and hydrothermally treated ZTA heads exceeded the minimum burst strength stipulated by the US Food and Drug Administration (FDA) despite severe test conditions. Lastly, Raman spectroscopic assessments of phase transformation and residual stresses on the fracture surface of the femoral heads were used to clarify burst-strength fluctuations and the effect of hydrothermal ageing on the material’s overall strength degradation.

## 1. Introduction

Since its commercial introduction in 2003, BIOLOX^®^delta (simply referred to as ZTA, henceforth) has rapidly achieved popularity as a bio-medical material for load-bearing components in artificial hip joints. ZTA has gradually replaced previous material generations of monolithic alumina, zirconia, and cobalt-chrome alloys [1,2]. Compared to other monolithic ceramics, ZTA possesses higher tensile strength and fracture toughness. Enhancements in both structural properties are due to a microscopic crack-tip shielding mechanism, which is induced by the tetragonal-to-monoclinic polymorphic transformation of partially stabilized zirconia dispersoids under tensile stress. This mechanism is commonly referred to as transformation toughening [3]. The fraction of zirconia (ZrO_2_) phase in ZTA also contributes to strengthening by suppressing abnormal grain growth in the alumina (Al_2_O_3_) matrix during sintering and by producing compressive residual stresses that compress its grain boundaries [4].

The polymorphic transformation in ZTA is also driven by environmental conditions [5]. This circumstance, usually referred to as low-temperature hydrothermal degradation (or ageing), has raised concerns about the in vivo microstructural stability of ZTA. When ZTA was first released to the orthopedic market, the maker claimed that the ceramic was fully stabilized and unaffected by the hydrothermal environment [6,7]. However, early literature on ZTA ageing kinetics indicated that the t→m transformation was sensitive to composition and processing [8]. An additional preliminary investigation showed that ZTA underwent hydrothermal ageing in autoclave at relatively low temperatures [9]. Successive systematic analyses showed that hydrothermally aged ZTA femoral heads suffered significant polymorphic transformation [5,10]. In response to these concerns, the maker provided predictions based on in vitro accelerated ageing tests that estimated appreciable in vivo transformation only after bodily exposure for hundreds of years [11]. These predictions were based on the model developed by Mehl-Avrami-Johnson [12] (i.e., one hour of hydrothermal ageing under 2 bar at 134 °C in steam was equivalent to ~3~4 years in vivo [13]). However, several independent research groups have subsequently reported that short- and mid-term ZTA femoral head retrievals exhibited significantly higher amounts of polymorphic transformation [14,15,16,17,18,19,20]. These experimental results raised serious concerns with respect to the validity of the American Society for Testing and Materials (ASTM) in vitro model [21]. In its current form, the model fails to predict the in vivo surface phase instability of hip joint components made of ZTA. Additionally, it has been reported that polymorphic transformation of zirconia is severely enhanced in proximity of metal staining [22,23] because the hydrogen generated from the reactions between metal and steam increase surface dehydroxylation of the alumina lattice, thus accelerating the flow of water molecules and free oxygen to vacancy sites in the zirconia lattice [23,24]. Finally, it was recently observed that ZTA degradation was exacerbated at the interface of metallic trunnions, where contact stresses reached their maximum due to micromotion. This resulted in fretting wear, metal transfer, cracks, and a strong increase in the fraction of monoclinic zirconia [25].

In this paper, we attempt to relate long-term hydrothermal ageing of ZTA femoral heads to their burst strength. Besides obtaining an independent evaluation of burst strength by sampling a statistically meaningful number of femoral heads randomly acquired from the marketplace, this study attempted to confirm previous studies by other investigators [11,26,27] which showed that the burst strength of ZTA femoral heads was not compromised by hydrothermal ageing to the extent that it failed to meet the 46 kN limit required by FDA for tests based on the ISO 7206 standard [28].

## 2. Materials and Methods

### 2.1. Samples

Twenty-four BIOLOX^®^delta ceramic femoral heads (CeramTec, Plochingen, Germany) were used in this study. All samples were Ø28 mm L type femoral heads and were randomly acquired from the marketplace in three different nations. According to their serial numbers, they all came from different batches, but were all produced in 2015. BIOLOX^®^delta is a patented Al_2_O_3_-matrix composite containing 17% ZrO_2_ (partially stabilized with ~1.3 mol% Y_2_O_3_), 3% SrAl_2_O_4_, and 0.6% Cr_2_O_3_ [17]. The presence of chromium dopant confers on the material a characteristic pink color. The consistency of the chemical composition and crystallographic structure of each femoral head was verified by X-ray Diffraction (XRD) and Raman spectroscopy before testing.

### 2.2. Hydrothermal Ageing

Hydrothermal ageing was performed in an autoclave at 132 °C under 2.0 bar pressure.

### 2.3. Burst-Strength Testing

**Figure 1:**
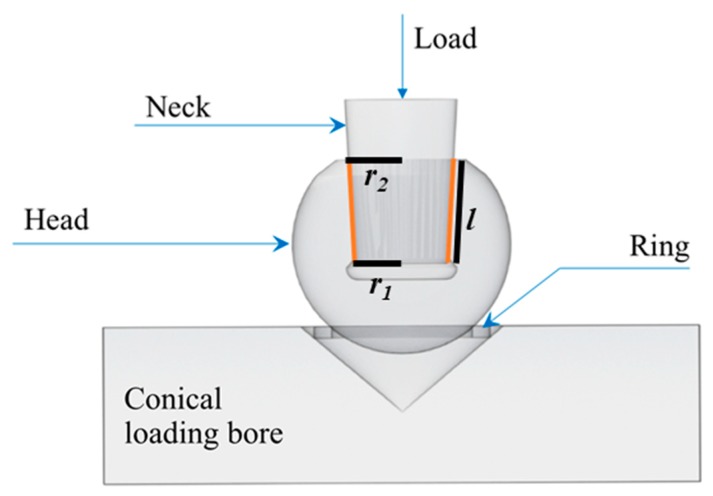
Schematic representation of the burst-strength testing equipment, following the international standard ISO 7206-10.

Samples were unpackaged and then compression tested to failure in burst-strength configuration using CoCr or Ti-6Al-4V trunnions and following the international standard ISO 7206-10 (Figure 1). The load, applied through a stem “neck”, was constantly increased at a rate of 0.5 kN/s until fracture. The samples were aligned by means of a conical loading bore (Brinell hardness between 150 and 250 HB) and a copper-centering ring. The following sets of samples were tested:

(1) Pristine heads (i.e., as received from the factory): two sets of n = 6 heads were simply unpackaged and then compression tested to failure using CoCr or Ti6Al4V trunnions.
(2) Moderately loaded and hydrothermally aged heads: n = 6 heads were repetitively loaded and unloaded at 3 kN and ~1 kN (maximum and minimum load respectively), respectively, onto CoCr trunnions followed by 15 h of autoclave ageing at 132 °C in the unloaded condition. This load-unload and autoclave cycle was repeated 10 times for a total of 150 h. The heads were then compression tested to failure using CoCr trunnions.
(3) Severely loaded and hydrothermally aged heads: n = 6 heads were repetitively loaded and unloaded at 20 kN and ~10 kN, respectively onto CoCr followed by 15 h of autoclave ageing at 132 °C in the unloaded condition. This load-unload and autoclave ageing cycle was then repeated 10 times for a total of 150 h. The test was further augmented by one final loading using CoCr trunnions with the inclusion of 0.5 mL of phosphate-buffered saline solution in the taper bore, followed by subjecting the assembled heads to a final 15 h autoclave cycle at 132 °C. These heads were then compression tested to failure using CoCr trunnions.

### 2.4. Sample Characterizations

#### 2.4.1. Laser Microscopy

Micrographs were taken using a 3D laser-scanning microscope (VKX200K series, Keyence, Osaka, Japan) with magnifications ranging from 10× to 150×, and numerical aperture between 0.30 and 0.95. The microscope used an automated x-y stage and an autofocus function for the z-range, enabling the acquisition of composite images over large portions of the samples, within a maximum z-range of 2 mm.

#### 2.4.2. Scanning Electron Microscopy

A field-emission-gun scanning electron microscope (JSM 7001F Scanning Electron Microscope, JEOL, Tokyo, Japan) was used to observe the fracture surface morphology of the femoral head samples. The instrument was equipped with an Electron Dispersive X-ray Diffraction (EDS) probe. All images were collected at an acceleration voltage of 10 kV and magnifications between 100× and 50,000×. All samples were sputter-coated (Cressington, Watford, UK) with a thin (20 to 30 Å) platinum layer.

#### 2.4.3. Raman Spectroscopy

Raman spectra were collected at room temperature by means of a triple monochromator (T-64000, Jobin-Ivon/Horiba Group, Kyoto, Japan) equipped with a charge-coupled device (CCD) detector. The spectra were analyzed by commercially available software (LabSpec, Hori-ba/Jobin-Yvon, Kyoto, Japan). The excitation source was a 532 nm Nd:YVO_4_ diode pumped solid-state laser (SOC JUNO, Showa Optronics Co. Ltd., Tokyo, Japan) operating with a nominal power of 200 mW. The lateral resolution of the Raman microprobe was on the order of 1 µm. Series of 250 µm × 250 µm square maps (composed of 21 × 21 data points each) were collected from each investigated area. The acquired spectra were then analyzed, and maps acquired at different locations merged to produce macroscopic maps covering large portions of the sample surface. In-depth profiles were also generated using one square map of the above size probed to a specific depth.

## 3. Results

### 3.1. Burst-Strength Test Results and Post Fracture Head Reconstruction

**Figure 2:**
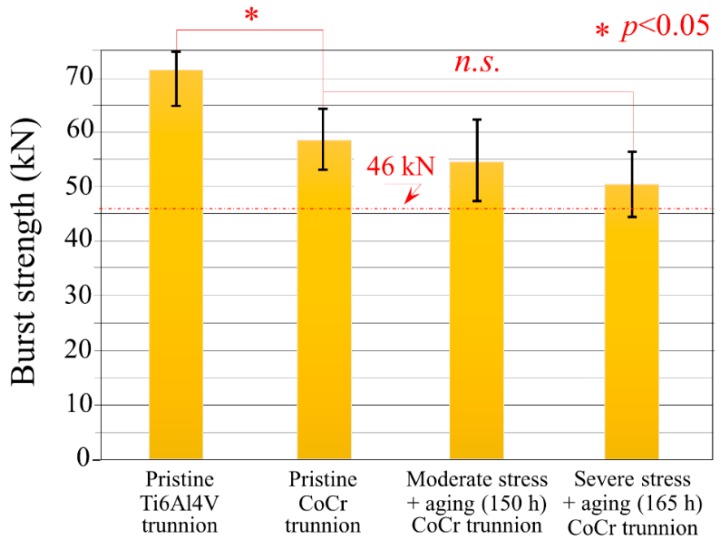
Burst-strength results obtained on the different series of samples.

Under moderate to severe test conditions, all the femoral head components met the FDA minimum average burst-test standard of >42 kN, with no failures below 20 kN (Figure 2). A significant difference in burst strength (with statistical significance using the Student’s *t*-test and a p-value of 0.05 for significance) was found between pristine heads tested against Ti6Al4V and CoCr trunnions, despite the common testing geometry and overall procedures.

Differences in burst-strength results obtained using Ti6Al4V and CoCr trunnions were due to differences in elastic modulus between the two alloys [29]. CoCr possesses a higher (almost double) modulus when compared with Ti6Al4V, meaning that the contact area between trunnion and femoral head under stress was smaller [30]. Even though higher rigidity was reported to reduce the amount of damage observed at the trunnion interface [30] on surfaces with comparable morphologies [31], the changes in contact pressure distribution led to higher stress intensification at the top of the trunnion which reduced the expected ultimate load during burst testing because the critical contact pressure was reached at lower applied loads [32].

Moderate and severe loading coupled with hydrothermal ageing affected the burst strength of components tested with CoCr trunnion with reductions in average loads of 6.2% and 16.4%, respectively. Using the Student’s *t*-test and a p-value of 0.05 for significance, the difference between the pristine heads and the moderately loaded and hydrothermally aged heads was not significant (p = 0.42); whereas the difference between the pristine heads and the severely loaded and hydrothermally aged heads was just beyond statistical significance (p = 0.053) (cf. Figure 2).

**Figure 3:**
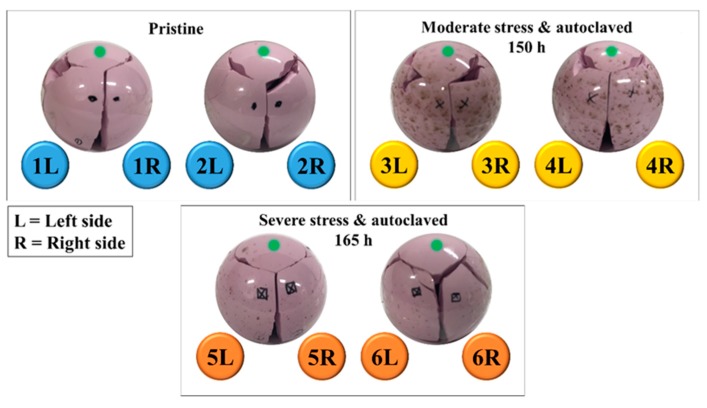
Reconstruction of two representative burst-strength-tested femoral heads tested against CoCr trunnions, for each of the respective testing conditions. The top of each femoral head has been marked with a dot.

**Figure 4:**
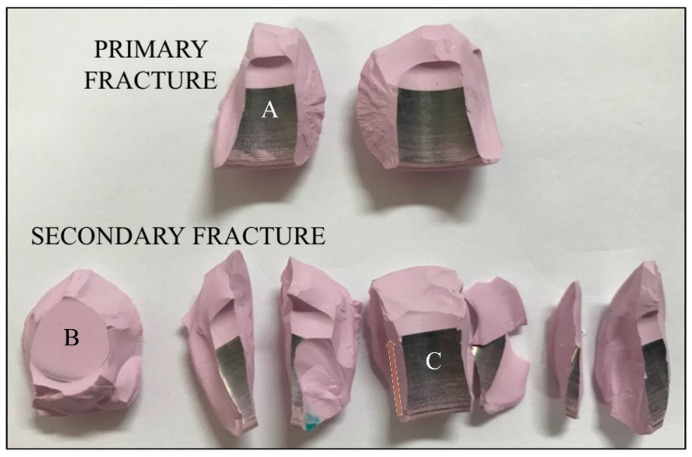
Main fragments of a burst-strength-tested ceramic femoral head, demonstrating primary and secondary fracture surfaces. A, B, and C mark a fragment with metallic stains, the top surface of the bore and some oriented marks, respectively.

The reconstruction of two representative burst-strength-tested femoral heads tested against CoCr trunnion is shown in Figure 3 for different testing conditions (cf. labels). All samples had a similar fracture morphology: (i) one large, almost circular fragment on the top of the ball; (ii) a few (4~9) large, longitudinal side fragments covering the area from the top fragment to the junction with the trunnion; and, (iii) a variable number of smaller fragments mostly with three types of morphology: (a) spike-like (where one of the dimensions was one order of magnitude larger than the other two), (b) blade-like (with a surface area higher than 1 cm^2^ but thickness below 0.5 mm), and (c) polyhedron-like (<3 mm in maximum dimension). All fragments were carefully collected after testing and classified into two main fractured parts showing the presence of a “mirror zone” (referred to as “primary fracture” parts) and the remaining parts (referred to as “secondary fracture” parts) (Figure 4). However, a complete reconstruction of the femoral head proved to be difficult. For all samples, up to 5% of the volume was lost in unidentifiable fragments.

Metallic stains were observed on all internal surfaces (cf. location labeled A in Figure 4). These stains completely covered about 4/5 of the cylindrical surface of the head cavity. The absence of stains on the top surface (cf. location B in Figure 4) indicates that the stem never reached the top of the head cavity during testing. The oriented scars clearly visible at the base of the head (cf. location C in Figure 4) were caused by the machining marks on the stem surface and their apparent color gradient (cf. arrow) is caused by increasing contact forces between the head and trunnion, which resulted in higher amounts of transferred metal.

### 3.2. Surface Fracture Morphology

**Figure 5:**
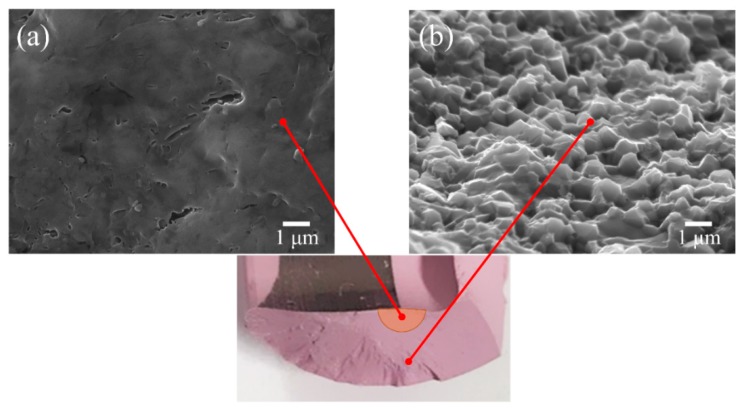
Scanning Electron Microscope images of two areas of the main fracture surface: (**a**) mirror-like and (**b**) intergranular.

Fracture surfaces were divided into two main regions, depending on their position and their mechanical behavior during mechanical testing. The portions of surface in the immediate neighborhood of the main fracture origin are henceforth referred to as “mirror-like areas”, due to their fully transgranular morphology. Conversely, fractured areas far away from the fracture origin and reaching the external surface were defined as “intergranular areas”; namely areas in which the fracture was fully intergranular. An example of mirror-like and intergranular areas are shown in Figure 5a,b, respectively. In the mirror-like area, secondary cracks and small, roundish porosity was detected. The latter defects formed during fracture due to zirconia grain pullout.

### 3.3. Zirconia Phase Transformation

**Figure 6:**
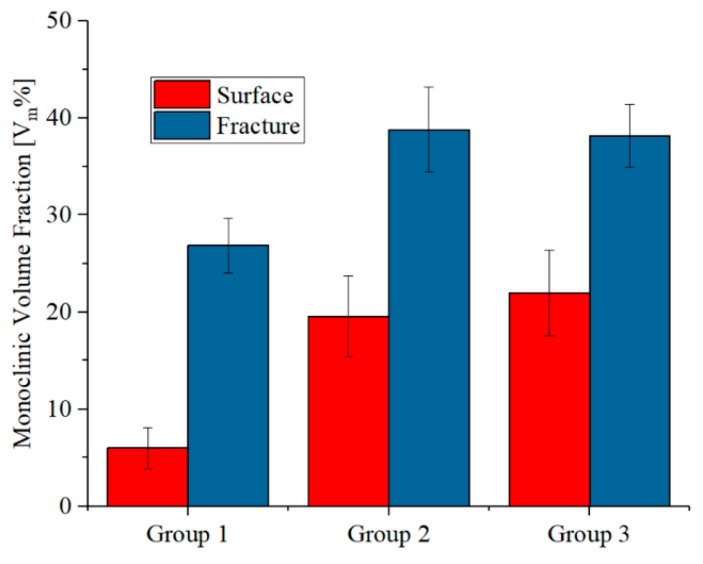
Monoclinic volume fractions as measured by Raman microprobe on the external surface (red bars) and on the fracture surface (blue bars) of the samples tested at different conditions.

Figure 6 shows the fraction of monoclinic zirconia inside the primary fracture surface and on the external surface as a function of the simulated ageing for CoCr trunnions. As expected, the decreased mechanical properties observed in Figure 2 corresponded to a variation in the fraction of monoclinic zirconia, which increased from about 6% to 23% on the external surfaces and from 27 to 37% on the primary fracture area. On both regions, the amount of monoclinic zirconia increased due to the thermal-mechanical ageing treatment, but the values acquired on the external surfaces of the femoral head appeared to be affected more as the increment was sensibly higher.

It must be observed that the amount of transformation obtained at the fracture area as compared to the pristine material was caused by the combination of three dependent factors: hydrothermal and mechanical ageing, mechanical load, and the energy release caused by the final burst-strength testing. The difference between the amount of monoclinic zirconia on the external surface (blue bars) and on the fracture area (red bars) reduced with exacerbated testing conditions, meaning that the transformation toughening mechanism of zirconia was losing its effectiveness.

### 3.4. Macroscopic Analysis

For both glasses and polycrystalline ceramics, there is a well-known correlation between ultimate strength and the size of the mirror region at fracture origins. As an example, Smekal et al. [33] proposed and refined the following relationship (1):

(1) $$\sigma \;R^{m}\;=\;A$$

where *σ* is the stress, R is the mirror radius and *m* and *A* are constants. A value of 0.5 was obtained for *m* in the case of annealed glasses [34], but it was also confirmed for fine-grained, strong polycrystalline alumina [35] and other ceramics [36], leading to the simplified Equation (2):

(2) $$\sigma \sqrt{R}\;=\;A$$

For imperfect circular mirror regions, the radius R can be approximated with the average medium radius ($R_{avg}^{M}$), calculated using Equation (3):

(3) $$R_{avg\;}^{M}=\;\frac{R_{1}\;+\;R_{2}\;+\;R_{d}}{3}$$

where $R_{1}$ and $R_{2}$ are the radiuses calculated on the two sides of the fracture origin and $R_{d}$ is the radius orthogonal to the external surface.

The applied stress cannot be precisely calculated at the trunnion of the femoral heads, as both contact areas and stress distributions are neither constant over time nor uniform across the surface. A rough estimation (σ’) can be obtained by considering the contact area at the trunnion interface from Figure 1 as Equation (4), and dividing the external applied load L by:

(4) $$\sigma^{\prime }\;=\;\frac{L}{A}\;=\;\frac{L}{\pi l(r_{1}\;+\;r_{2})}$$

where $r_{1}$ and $r_{2}$ are the upper and lower radius of the conical interface and l is the length of the contact region. Pressure is not constant along the length, as can be clearly observed by the amount of metal transfer occurred during testing. Assuming a parabolic stress distribution with a minimum at the lower radius and a maximum about 5 times higher than the minimum [37,38,39], a more accurate σ value of local stress to be used in the regression calculation of the residual stresses can be assumed by “redistributing” the previous estimation (σ’) in a parabolic fashion along l with the conditions that $\sigma \left(r_{1}\right)\;=\;5\sigma \left(r_{2}\right)$.

**Figure 7:**
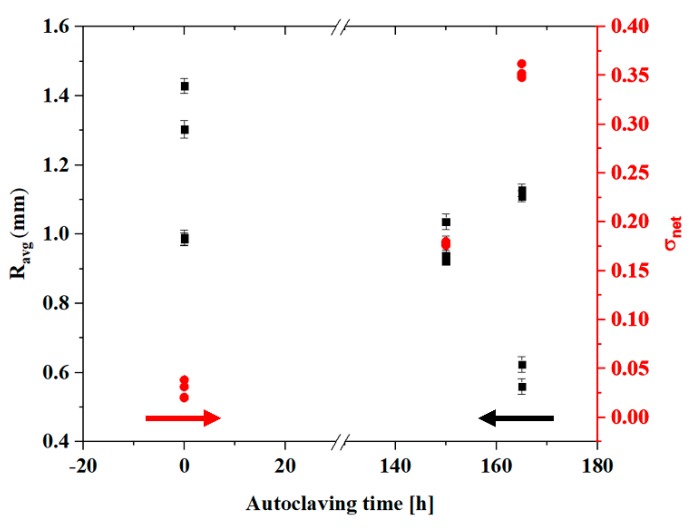
Radius of the mirror region and residual stresses as a function of the ageing conditions, for CoCr trunnions (red circles for σ, black squares for R_avg_).

Figure 7 shows the radius of the mirror region and the $\sigma_{net}$ residual stress as a function of ageing conditions for CoCr trunnions. It was observed that the radius values a large statistical dispersion. Nevertheless, the distribution of residual stresses followed a clear trend where pristine samples had values close to zero and autoclaved samples were on the order of hundreds of MPa. Even if the calculations are affected by a large degree of approximation, the trend can be considered representative for the relationship between ageing and residual stresses at the fracture surface.

### 3.5. Microscopic Evaluations

**Figure 8:**
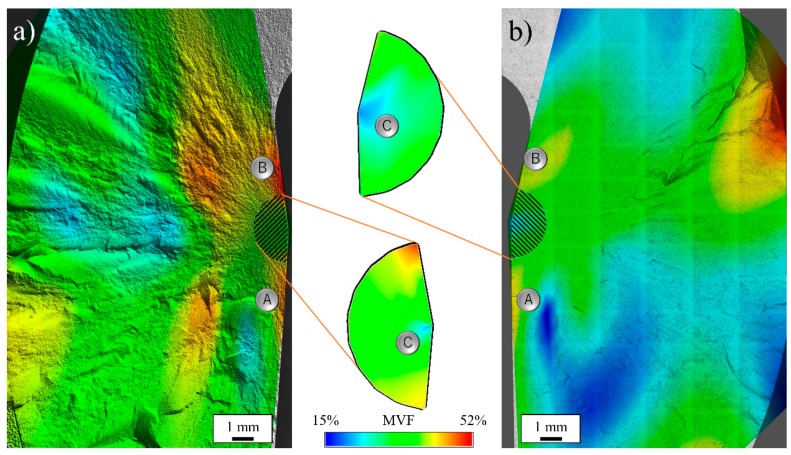
Monoclinic volume fraction (MVF) as measured by Raman spectroscopy using the Katagiri equation, as a function of portion on the primary fracture surface for a (**a**) Group 2 and a (**b**) pristine femoral head tested against CoCr trunnions.

Figure 8 shows the fraction of monoclinic zirconia as measured by Raman spectroscopy using the Katagiri equation [40], as a function of position on the primary fracture surface for a moderately loaded and hydrothermally aged (Group 2) (a) and a pristine (Group 1) (b) femoral head tested against CoCr trunnions. It was observed that the amount of induced t→m transformation was higher for the hydrothermally aged sample as previously reported by many authors.

Two regions of relatively high transformation were observed on the two sides of the mirror zone of both samples. While high transformation in the trunnion contact region (A) was caused by contact mechanics (compressive), the area in (B) was not subject to direct loading and represents a region of high flexural stress, as deducted from the shape of the fragments (Figure 3 and Figure 4). As the contact stress at the trunnion is perpendicular to the internal surface while the applied load pushes the ball from the top, the ceramic ball is forced to expand radially to compensate. The fulcrum of this momentum is in in the surroundings of B, meaning that in that region tetragonal zirconia transformed due to the mechanical (tensile) stresses alone.

The region of relatively low transformation close to both (A) points indicates that the contact stress only influences the first millimeter of material and does not penetrate deeper inside the monolithic component.

In both Group 1 and Group 2 components the mirror regions have relatively small fractions of monoclinic zirconia, with a point of minimum at the center of the mirror (C), where the fractures were generated.

### 3.6. Residual Stresses

The amount of residual stress stored inside the crystallographic structure of the composite ceramic femoral heads was estimated using Raman spectroscopy by measuring the shift of specific bands and multiplying the value by their piezo-spectroscopic coefficient. The cumulative, net residual stress ($\langle \sigma_{\mathrm{net}}\rangle$) was then calculated using the Equation (5):

(5) $$\langle \sigma_{\mathrm{net}}\rangle \;=\;V_{a}\langle \sigma_{a}\rangle \;+\;V_{z}\left[V_{m}\langle \sigma_{m}\rangle \;+\;\left(1\;-\;V_{m}\right)\langle \sigma_{t}\rangle \right]$$

where $\langle \sigma_{a}\rangle$, $\langle \sigma_{m}\rangle$ and $\langle \sigma_{t}\rangle$ are the stresses measured on the alumina, monoclinic and tetragonal zirconia phases, $V_{a}$ is the fraction of alumina in the composite material, $V_{z}$ is total fraction of zirconia and $V_{m}$ is the relative amount of monoclinic zirconia. It must be noted that while the amount of alumina $\langle \sigma_{a}\rangle$ is constant over time and across the sample volume, the amount of monoclinic and tetragonal zirconia needs to be addressed locally before calculating the cumulative net stress.

**Figure 9:**
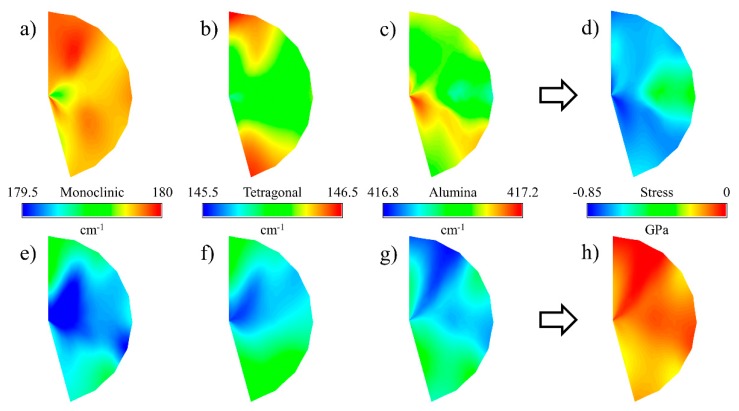
Raman band shifts for monoclinic zirconia, tetragonal zirconia and alumina and equivalent stress on Group 2 (**top row**) and pristine (**bottom row**) femoral head.

The Raman shifts of each representative phase peak for a moderately loaded and 150 h autoclaved sample (Group 2) are reported in Figure 9a–c, along with the calculated $\langle \sigma_{\mathrm{net}}\rangle$ Figure 9d. It was observed that band positions are usually higher than the reference values, resulting in compressive residual stresses as high as 850 MPa. The area around the center of the mirror region, where the crack originated, reaches the highest values of residual stresses. In the case of a reference, pristine femoral heads (Group 1) Figure 9e–h, band shifts are usually lower than the hydrothermally aged samples, resulting in residual stresses close to zero. Surprisingly, the fracture origin at the center of the mirror region showed the lowest amount of residual stress.

**Figure 10:**
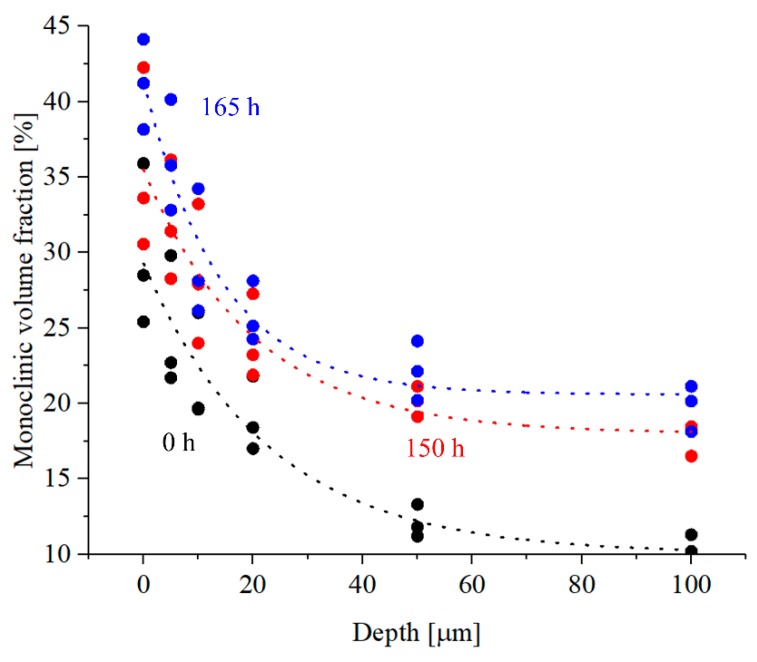
In-depth profile of monoclinic volume fraction as a function of hydrothermal ageing, as measured by Raman spectroscopy using the Karagiri equation.

Figure 10 shows the in-depth profiles of monoclinic volume fraction and residual stresses for a Group 1, Group 2, and a Group 3 femoral head, as measured in three randomized locations of the mirror region. It was observed that the values of monoclinic volume fraction rapidly decreased, reaching a stable value after about 50 μm. The values obtained for the aged sample are higher, but with a comparable statistical distribution. For residual stresses, the trend is opposite, with higher values in-depth that slowly decreased until reaching the fracture surface. Additionally, residual stresses were always higher for the pristine head when compared to the hydrothermally aged sample.

## 4. Discussion

It is noteworthy that despite these moderate to severe test conditions, all of these components met the FDA minimum average burst-test standard of >46 kN, with no failures below 20 kN [21]. Moderate (Group 2) and severe (Group 3) loading coupled with hydrothermal ageing affected the burst strength of these components with reductions in average loads of 6.22% and 16.44%, respectively. Using the Student’s *t*-test and a p-value of 0.05 for significance, the difference between the pristine heads (Group 1) and the moderately loaded and hydrothermally aged heads (Group 2) was not significant (i.e., p = 0.42); whereas the difference between the pristine heads (Group 1) and the severely loaded and hydrothermally aged heads (Group 3) was just beyond statistical significance (i.e., p = 0.053). The reported average burst-test data from CeramTec for pristine heads of this geometry is 83 kN [11]. This same value was again reported in a separate paper published in 2012 [41]. Values of ~80 kN were also reported in a study by Corfield et al., with retention of this strength after 50 h of hydrothermal ageing [42]. In the most recent publication by Garino, the burst strength of BIOLOX^®^delta was reported to be 101 ± 7 kN. However, no details on the geometry of these heads were provided in the report [43]. Marketing literature for BIOLOX^®^delta also touts these high burst strengths [44]. Two materials are typically used—A titanium alloy (TI6Al4V) and a cobalt-chromium alloy (CoCr). Literature also shows some lower burst-test results (around 70 kN) for components subjected to hydrothermal ageing [42]. Perhaps these parts were tested using CoCr trunnions.

Values reported in the literature for pristine ZTA femoral heads of the same diameter are at least 10% [45] higher than the ones shown in Figure 2 for CoCr trunnions. Similar discrepancies in burst testing have usually been associated with stress intensification at the taper interface. For short-neck heads engage the taper over a larger surface area thereby reducing stress, while in long–neck configurations, stress transfer occurs lower in the taper in an area that is weaker [46]. However, even small contaminants on the taper were reported to influence burst-test results by altering the stress distribution [47].

Moreover, previous literature reported that the burst strength of ZTA was unaffected by hydrothermal ageing up to 20 h [48] or even 50 h [42]. The values of ultimate load measured at 150 and 165 h of loading and hydrothermal ageing treatment (Figure 2) suggest that small losses were already present even at 20 and 50 h, but the amount of loss could not be measured precisely due to the large scatter within the data points. The fraction of monoclinic zirconia grows linearly over hydrothermal ageing and load testing, in a similar fashion as previous literature research [5].

The decrease in the radius of the fracture mirror with hydrothermal ageing time is a clear signal that residual stresses are stored inside the microstructure. The composite ceramic loses the ability to “absorb” mechanical stress and this effect negatively influences the mechanical performance.

Raman localized analyses showed that the amount of monoclinic zirconia in the mirror areas was sensibly lower than in the surrounding regions, in particular when compared to the taper contact areas (Figure 8a,b A) and the point of maximum bending stress (Figure 8a,b B). This behavior indicates that the tetragonal-to-monoclinic transformation was influenced by the crack propagation speed and only a limited amount of transformation occurred during transgranular fracture. Moreover, the fracture origin at the center of the mirror (C) registers the lowest amount of monoclinic zirconia.

The mirror areas of autoclaved samples appeared to be under high compressive stress, while the pristine references had values of stress close to zero (Figure 9). It can be assumed that most of the stress was dissipated into crystallographic transformation, as previously reported in the literature (i.e., an increasing fraction of monoclinic phase pushes the residual hydrostatic stress field to the tensile side [5]). For the autoclaved samples, the highest amount of monoclinic zirconia resulted in greater residual stress as the microstructure was not able to absorb the same amount of energy.

It was observed that the in-depth profiles of the fracture areas show similar levels of residual stress after about 50 μm, while in the region close to the surface higher residual stress was registered for the hydrothermally aged samples; meaning that part of the mechanical stress was converted into crystallographic deformation.

## 5. Conclusions

The results of these tests suggest that BIOLOX^®^delta continues to meet FDA requirements for minimum strength regardless of the severity of repetitive stress and hydrothermal ageing. However, the acquired data markedly differ from other reported literature values. This difference is likely due to the use of Ti6Al4V versus CoCr test trunnions. Our original alternative hypothesis was disproved. We expected to see a marked reduction in the burst strength of BIOLOX^®^delta based on a significant loss of surface fracture toughness [49]. However, this was not the case. BIOLOX^®^delta retains its strength despite severe hydrothermal testing.
